# Supplementary material for: Adversarial robustness assessment: Why in evaluation both L0 and L∞ attacks are necessary
Source: PLoS One. 2022 Apr 14;17(4):e0265723. doi: 10.1371/journal.pone.0265723 (PMC9009601; doi:10.1371/journal.pone.0265723)
Supplement: Attachment — Submitted filename: Rebuttal - Threshold.pdf [file pone.0265723.s001.pdf]

Dear Academic Editor and Reviewers,

We thank the reviewers for their constructive feedback on our article. We have revised our article according to the feedback to improve the article and below is our detailed response to the reviews and journal requirements.

Reviewer #2: The authors must carefully check the language and English grammar. There are also some typos.

The article is revised and has been corrected for typing and grammatical errors.

Reviewer #2: Why the Authors have stated that the effectiveness of the L_0 and L_∞ attacks?. An additional explanation would be helpful for potential readers.

Thank you for pointing it out. To improve the clarity on the topic, our abstract is revised to state explicitly that the effectiveness of L_0 and L_∞ attacks used in the article highlight the fact that neural networks are vulnerable to either one or both types of attacks, even when equipped with adversarial defences. Therefore, it is essential to evaluate both types of attacks.

Reviewer #3: Some sentence throughout the paper are strange which make the paper lack readability. Author should check and correct it. There are some typos and grammatical errors. The authors should check carefully and correct seriously. There are too many alignment issues. Authors should correct it.

The readability of the article is improved with simpler sentence structures and also has been corrected for typing and grammatical errors.

Reviewer #3: How to construct the derivation (2), need detailed explanations.

We appreciate your feedback on pointing this out. To improve the clarity on the understanding of the derivation, further details have been updated in the text. The derivation of (2) follows from the definition of adversarial samples that, adversarial samples have a different classification than the original sample, thus effectively inducing misclassification.

Reviewer #3: The main contributions of the paper should be clearly stated.

In order to highlight and clearly state our contributions in this article according to the feedback, the paragraph title "Contributions" is added to the content where the contributions are discussed.

Reviewer #3: Discuss the technical difficulty in dealing with proposed scheme.

The technical difficulty and limitations of the proposed scheme are now discussed in the revised section "Guaranteeing the Quality of Adversarial Samples".

Reviewer #3: One or two remarks should be given. According to the topic of the paper, the authors should discuss some interesting problem as future work in conclusion, such as 10.1109/ACCESS.2021.3123058; 10.1016/j.jfranklin.2020.01.016; 10.1109/ACCESS.2021.3060044.

Our conclusions are revised to provide a few remarks and discuss interesting future research directions which could be pursued.

Journal Requirement: Please ensure that your manuscript meets PLOS ONE's style requirements, including those for file naming.

The manuscript is checked and it is ensured that the manuscript follows PLOS ONE's style requirement.

Journal Requirement: Please remove any funding-related text from the manuscript and let us know how you would like to update your Funding Statement. Currently, your Funding Statement reads as follows:
"DV, JST-Act-I Grant Number JP-50243, https://www.jst.go.jp/kisoken/act-i/index.html
DV, JSPS KAKENHI Grant Number JP20241216, https://www.jsps.go.jp/english/e-grants/"
Please include your amended statements within your cover letter; we will change the online submission form on your behalf.

Funding-related text is removed from the manuscript. Further, we would like our funding statement to be read as follows,

"*Danilo Vasconcellos Vargas, JST-Act-I Grant Number JP-50243,*
https://www.jst.go.jp/kisoken/act-i/index.html

Danilo Vasconcellos Vargas, JSPS KAKENHI Grant Number JP20241216,
https://www.jsps.go.jp/english/e-grants/"

Kindly amend the statements in the submission.

Journal Requirement: Please review your reference list to ensure that it is complete and correct.

The reference list was reviewed and none of the references are retracted.

Yours faithfully

Shashank Kotyan
Danilo Vasconcellos Vargas
